# MORE-RNAseq: a pipeline for quantifying retrotransposition-capable LINE1 expression based on RNA-seq data

**DOI:** 10.3389/fbinf.2025.1575346

**Published:** 2025-05-22

**Authors:** Yutaka Nakachi, Jianbin Du, Risa Watanabe, Yutaro Yanagida, Miki Bundo, Kazuya Iwamoto

**Affiliations:** ^1^ Department of Molecular Brain Science, Graduate School of Medical Sciences, Kumamoto University, Kumamoto, Japan; ^2^ Department of Geriatric Psychiatry, The Affiliated Mental Health Center of Jiangnan University, Wuxi, China; ^3^ Department of Psychiatry, Icahn School of Medicine at Mount Sinai, New York, NY, United States

**Keywords:** LINE-1 (L1), expression, RNA-seq, retrotransposon, transcription, cancer, aging

## Abstract

Retrotransposon long interspersed nuclear element-1 (LINE-1, L1) constitutes a large proportion of the mammalian genome. A fraction of L1s, which have no deleterious mutations in the structure, can amplify their copies via a process called retrotransposition (RT). RT affects genome stability and gene expression and is involved in the pathogenesis of many hereditary diseases. Measuring expression of RT-capable L1s (rc-L1s) among the hundreds of thousands of non rc-L1s is an essential step to understand the impact of RT. We developed mobile element-originated read enrichment from RNA-seq data (MORE-RNAseq), a pipeline for calculating expression of rc-L1s using manually curated L1 references in humans and mice. MORE-RNAseq allows for quantification of expression levels of overall (sum of the expression of all rc-L1s) and individual rc-L1s with consideration of the genomic context. We applied MORE-RNAseq to publicly available RNA-seq data of human and mouse cancer cell lines from the studies that reported increased L1 expression. We found the significant increase of rc-L1 expressions at the overall level in both inter- and intragenic contexts. We also identified differentially expressed rc-L1s at the locus level, which will be the important candidates for downstream analysis. We also applied our method to young and aged human muscle RNA-seq data with no prior information about L1 expression, and found a significant increase of rc-L1 expression in the aged samples. Our method will contribute to understand the role of rc-L1s in various physiological and pathophysiological conditions using standard RNA-seq data. All scripts are available at https://github.com/molbrain/MORE-RNAseq.

## Introduction

Long interspersed nuclear element-1 (LINE-1, L1) is the most representative class of retrotransposons in the mammalian genome, representing 17% of the human (Lander et al., 2001) and 19% of the mouse (Waterston and Lindblad-Toh, 2002) genomes. In the human genome, there are more than 600,000 L1 copies, and approximately 5,000 L1s are 6 kb-long, full-length L1s. Full-length L1 includes two protein coding regions called open reading frame 1 (ORF1) and ORF2. ORF1 encodes an RNA-binding protein, and ORF2 encodes a protein that has endonuclease and reverse-transcriptase activities. Among them, approximately 150 L1s harbor no deleterious mutations in the structure (Pickeral et al., 2000; Ostertag and Kazazian, 2001; Kazazian, 2000), and can amplify L1 copies by a process called retrotransposition (RT) (Beck et al., 2011). In humans, the RT capable L1s (rc-L1s) mostly belong to the youngest L1 subfamily, Hs. L1 has evolved in a species-specific manner. In mouse genome, there are more than 9,000 full-length L1 copies. Among them, about 2,800 L1s are considered to be rc-L1s and they composed of several active subfamilies (Sookdeo et al., 2013). Additionally, in mice, there are mouse-specific repeat tandems, called monomers, at the upstream region of the 5′UTR (Adey et al., 1991).

The rc-L1s can autonomously retrotranspose in the mammalian genome. RT affects genome stability, gene structure, and gene expression and is often identified as the cause of many heritable diseases. In addition, the L1 ORFs are required for RT of other classes of retrotransposons, such as Alu and SVA in human. Accumulating evidence further suggests that increased activity of L1s in somatic cells is involved in the aging, inflammation, and pathophysiology of neuropsychiatric disorders (St Laurent et al., 2010; De et al., 2019; Simon et al., 2019; Bundo et al., 2014; Watanabe et al., 2023).

Measuring the activity of rc-L1s is therefore particularly important, and provides the insights into the molecular physiology and pathophysiology of the disease. Typically, qPCR targeting conserved L1 regions was used to quantitate their transcription level. Although convenient, as both a rare fraction of rc-L1s and major fraction of non rc-L1s are amplified together, this method has disadvantages in the specificity and resolution. Several bioinformatic procedures have been developed to assess L1 expression from next-generation sequencing-based data (Jin et al., 2015; Streva et al., 2015; Deininger et al., 2017; Lerat et al., 2017; Criscione et al., 2014; Yang et al., 2019; Ansaloni et al., 2022). Typically, their references of L1s were RepeatMasker data (http://www.repeatmasker.org/). However, lack of detailed curation resulted in reduced accuracy due to the incompleteness of the L1 definition in the database as well as the intrinsic complexity of L1s. For example, full-length L1 entries are often divided into several subregions, and some L1s are not identified in RepeatMasker. In addition, L1s often contain repetitive regions at their 5′ and 3′ ends, such as monomers at the 5′ ends in mice and around polyA signals at the 3′ ends in both human and mouse. Therefore, simple use of L1 entries causes erroneous mapping, such as multimatch and/or false-negative mapping results. In addition, several procedures do not distinguish expression between non-rc-L1s and rc-L1s, or do not estimate expression of rc-L1s at the single locus level. Technically, some procedures involve separate expression analysis of conventional genes and L1s, requiring additional procedures for direct comparison.

To address these shortcomings, we developed mobile element-originated read enrichment from RNA-seq data (MORE-RNAseq), a pipeline for quantitative analysis of rc-L1s. In MORE-RNAseq, sequence reads are mapped to the reference consisting of genes and manually curated rc-L1s. It is applicable to standard human and mouse short-read RNA-seq data with a few simple parameter adjustments and allows for simultaneous quantification of expression of genes and overall or individual rc-L1s.

## Methods

### Preparation of the curated L1 reference

Chromosomal locations of rc-L1s in humans and mice were acquired from L1Base 2 (Penzkofer et al., 2017). All L1 sequences were then manually curated, and L1-specific regions were chosen as the references. Repetitive sequence regions such as monomers at 5′termini in mice, conserved poly-A signals and the A stretch at 3′termini were excluded from the L1 reference to avoid artificial results from reads other than L1-originated reads. The curated rc-L1 reference and the gene annotation related to GRCh38/GRCm38 (Ensembl release 102) were included in the MORE references. Intergenic/intragenic annotations of rc-L1 were also based on the same Ensembl data. All information and annotation files are available on GitHub (https://github.com/molbrain/MORE-reference). This study also used the MORE reference as the TE GTFs (option '--TE’) with the GTF of normal genes (option '--GTF’) in TEtranscripts (Jin et al., 2015) analysis.

### Implementation of MORE-RNAseq

All scripts in the MORE-RNAseq pipeline and the Dockerfile including required tools are available at the GitHub site (https://github.com/molbrain/MORE-RNAseq). MORE-RNAseq involves a series of steps written by the ZSH (v5.0.2) shell script supported by CentOS7/8/Rocky or other Linux distributions. Some scripts rely on Perl5 (v5.16.3) and Java (v1.8.0), and installation of fastqc (v0.11.8), ea-utils (v1.01), Cutadapt (v1.18), Trimmomatic (v0.38), STAR (v2.6.0c), Samtools (v1.11) and RSEM (v1.3.3) should be required to use MORE-RNAseq. For visualization of the results with our workflow, downloading the relevant packages in the R language (v3.5.1) is needed. The version numbers of each tool shown above in brackets are those we used to develop and verify the pipeline.

### Application of MORE-RNAseq

We examined two human RNA-seq datasets [GSE100751 (Ansaloni et al., 2022) and GSE159217 (Penzkofer et al., 2017)] and one mouse RNA-seq dataset [GSE217036 (Guler et al., 2017)] to validate the MORE-RNAseq.

## Results

### Curation of rc-L1s and the workflow of MORE-RNAseq

**FIGURE 1:**
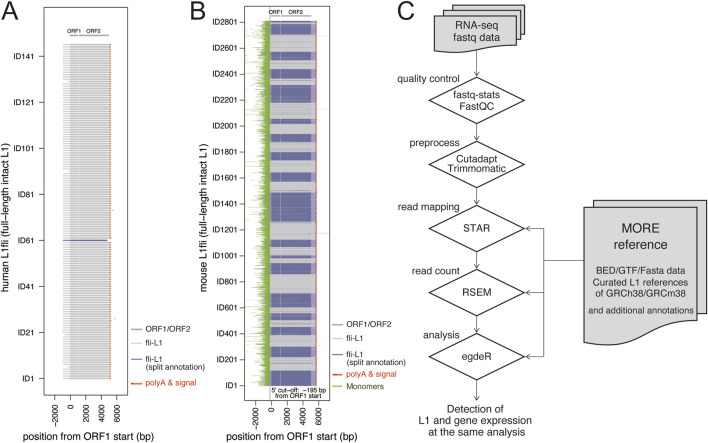
MORE reference and MORE-RNAseq workflow. **(A,B)** Alignment of all rc-L1s (full-length intact L1s) used as MORE references for humans **(A)** and mice **(B)**. In the plots, all L1s are aligned with the ORF1 start position as zero. The IDs of L1 are the same as L1Base2 entries. The L1 entries required for additional curations are shown by blues (Supplementary Figure S1). **(C)** The typical workflow of the MORE-RNAseq pipeline. The RNA-seq data in the fastq format from local labs or public data can be used. Quality control was performed on the raw data, followed by removal of adaptor and low-quality reads, and then alignment was performed to the reference genome. After alignment, expression values were calculated corresponding to the GTF data, and a dataset of TPM values was generated as a profile of the transcriptome, including L1s and other genes. The obtained results were imported into R for visualization and statistical analysis.

We retrieved rc-L1s in humans (N = 146) and mice (N = 2,811) using L1Base 2 (Penzkofer et al., 2017), followed by manual curation of all L1 sequences. Since some L1s are divided into multiple entries with different annotations or are only partially present in RepeatMasker, and given that nearly all rc-L1s are located within repetitive surrounding sequences, the reference regions of rc-L1s were curated and selected carefully (Supplementary Figure S1). For human rc-L1s, we defined the reference region from the 5′termini of the L1 entries to the nearest polyA signal downstream of each ORF2. For mice, we applied a 5′ cutoff at −195 bp upstream of the ORF1 start site to exclude the monomer repeat region and a 3′ cutoff at 665 bp downstream of the ORF2 end to avoid incorrect mapping of reads derived from other repetitive sequences other than L1. The selected L1 sequences are shown in Figure 1A,B. They were compiled into fasta, bed, and gtf format data for MORE-RNAseq, and are available on GitHub at https://github.com/molbrain/MORE-reference. The workflow of MORE-RNAseq is the same as for general RNA-seq analysis, with only the exchange from conventional GTF files for RNA-seq analysis to new GTF files, which include both the curated rc-L1s and general genes (Figure 1C).

### Case study results

To validate the reliability of MORE-RNAseq, we examined two human RNA-seq datasets [GSE100751 (Guler et al., 2017) and GSE159217 (Lagerwaard et al., 2021)] and one mouse RNA-seq dataset [GSE217036 (Novototskaya-Vlasova et al., 2022)].

**FIGURE 2:**
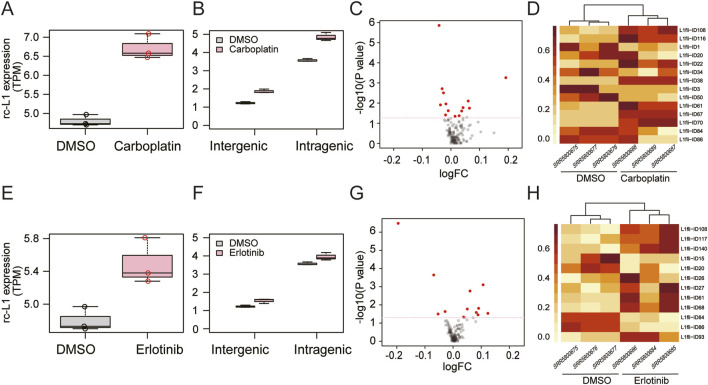
Application of MORE-RNAseq to human RNA-seq data (GSE100751). PC9 cells were treated with carboplatin **(A–D)** or erlotinib **(E–H)**. **(A,E)** Increased expression of overall rc-L1s in the carboplatin (P = 0.004 in Welch’s t-test) and erlotinib (P = 0.032) groups compared to the DMSO group. **(B,F)** Genomic context analysis. Carboplatin treatment led to significantly increased expression of intergenic (P = 0.002) and intragenic (P = 0.005) rc-L1s compared to the DMSO group. Erlotinib led to a significant tendency toward increased expression of intergenic (P = 0.034) and intragenic (P = 0.075) rc-L1s, respectively. **(C,G)** Volcano plots of rc-L1s. rc-L1s showing P < 0.05 in Welch’s test are indicated in red. **(D,H)** Heatmaps of differential expression of individual rc-L1s based on normalized values.

First, we examined the GSE100751 data (Figure 2) from a study that reported increased L1 expression in PC9 human cancer cells in response to carboplatin or erlotinib (Guler et al., 2017). In the previous study (Guler et al., 2017), L1 expression was analyzed with Salmon software (Patro et al., 2017) at the L1 subfamily level. We successfully confirmed increased expression of overall rc-L1s (sum of the expression of all rcL1s) by MORE-RNAseq (Figures 2A,E). In addition, we found increased expression in intragenic and intergenic rc-L1s (Figures 2B,F) and identified differentially expressed individual rc-L1s (Figures 2C,D,G,H).

We compared the results obtained from MORE-RNAseq with those from TEtranscripts (Jin et al., 2015), one of the widely used tools for quantifying all L1 expressions. In addition to using the default GTF reference file of TEtranscripts, we included the GTF reference file from MORE-RNAseq following the instructions provided. Since the downstream analyses of MORE-RNAseq and TEtranscripts differ, we compared the expected counts. As expected, TEtranscripts detected a significant increase in overall L1 expression levels, as well as rc-L1 expressions, when using the MORE reference (Supplementary Figure S2). Both methods demonstrated high concordance between the expected counts of rc-L1s from MORE and those of all L1s from TEtranscripts (R^2^ = 0.955). Similarly, a high concordance was observed between the expected counts of rc-L1s from MORE and those from TEtranscripts using the MORE reference (R^2^ = 0.941).

**FIGURE 3:**
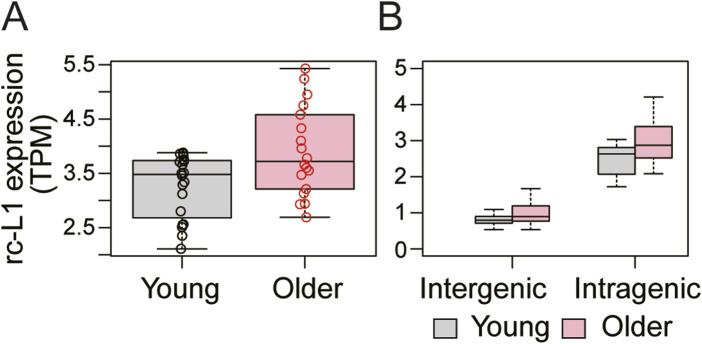
Application of MORE-RNAseq to human skeletal muscle RNA-seq data (GSE159217). **(A)** Overall rc-L1 expression levels were significantly increased in older skeletal muscle (P = 0.036, Welch’s t-test). **(B)** Both intergenic (P = 0.041) and intragenic rc-L1s (P = 0.011) showed significant increases.

Previous studies have suggested that L1 expression increases with cellular senescence or tissue aging (De et al., 2019; Lagerwaard et al., 2021; Novototskaya-Vlasova et al., 2022; Patro et al., 2017; Kumari and Jat, 2021). Based on this, we selected skeletal muscle RNA-seq data for young (19–25 years) and old (65–71 years) people (GSE159217) (Lagerwaard et al., 2021), with no prior information about L1 expression. We found that expression of overall rc-L1s was significantly increased in the skeletal muscle cells of older people compared to younger people (Figure 3A). We also found significantly increased expression of both intergenic and intragenic rc-L1s (Figure 3B).

**FIGURE 4:**
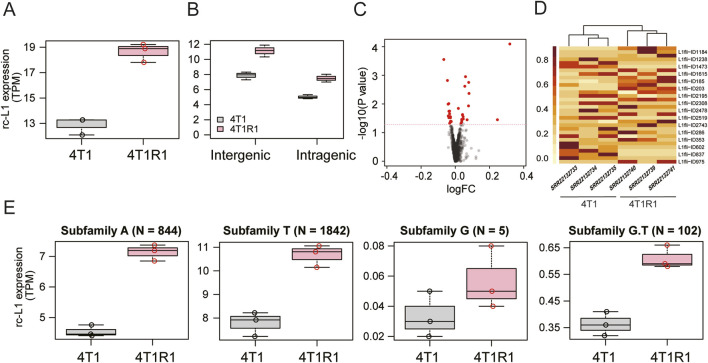
Application of MORE-RNAseq to mouse RNA-seq data (GSE217036). Comparison between 4T1 mouse cancer cells and cells with acquired 17-DMAG resistance (4T1R1). **(A)** Increased expression level of overall rc-L1s in 4T1R1 cells (P < 0.0001 in Welch’s t-test). **(B)** Genomic comparison. Both intergenic (P = 0.006) and intragenic rc-L1s (P = 0.005) showed significant increases. **(C)** Volcano plot of individual rc-L1s. rc-L1s showing P < 0.05 in Welch’s t-test are indicated in red. **(D)** Heatmap of differentially expressed rc-L1s based on normalized values. **(E)** Expression level at the subfamily level. Subfamilies except G (P = 0.199) showed significantly increased expression of rc-L1s (P < 0.005).

To verify the applicability of MORE-RNAseq to mouse RNA-seq data, we selected the RNA-seq dataset (GSE217036) from a study that reported increased expression of retroelements, including L1, in 4T1 mouse cancer cells that acquired chemical resistance (4T1R) (Novototskaya-Vlasova et al., 2022). In that study (Novototskaya-Vlasova et al., 2022), L1 expression was analyzed using TEtranscripts (v2.2.3) (Jin et al., 2015). We confirmed the increased expression of overall rc-L1s (Figure 4A), observed elevated levels of both intergenic and intragenic rc-L1s (Figure 4B), and identified differentially expressed individual rc-L1s (Figures 4C,D). Additionally, increased expression was noted at the subfamily level, except for subfamily G (Figure 4E).

## Discussion

Rapid and efficient expression analysis of rc-L1s from massive RNA-seq data is of significance to develop biomarkers and understand the pathophysiology of diseases. By using the curated rc-L1 reference, MORE-RNAseq enables estimation of expression of overall, subfamily, and individual rc-L1s. In our case study, MORE-RNAseq successfully detected increased expression of rc-L1s, including a case without prior information on L1 expression. In addition, genomic context analysis provided insights into the possible molecular mechanisms underlying altered expression of rc-L1s. For example, altered expression at the intergenic level suggests dysregulation of L1 expression mechanisms, whereas altered expression at the intragenic level may involve dysregulation of expression of nearby genes. Analysis of individual rc-L1s will provide important candidates for further biological study. For instance, five individual intragenic rc-L1s were commonly identified in PC9 cells in response to carboplatin or erlotinib (Figure 2). It should be noted that increased expression of overall rc-L1s did not always indicate increased expression of all individual rc-L1s. In our case study, differentially expressed individual rc-L1s showed both directions of change in all cases.

For genomic context analysis, we utilized only intergenic or intragenic information in this study. However, our GTF files and L1-related information consist of detailed annotations based on not only L1s but also locational relationships with nearby genes. Therefore, more precise analysis, such as the consideration of L1 orientation, could be conducted.

In the MORE-RNAseq analysis, we created references using only rc-L1s. Therefore, it is possible that sequence reads from non-rc-L1, such as full-length nonintact L1s, were mapped to the references. However, multimapped reads are processed as expected values by averaging the number of matching sites with STAR and RSEM, and the effect is alleviated. For L1 expression estimation with previous approaches, the sequencing library needs to be constructed with a specific method, and the analytical pipeline also should be adopted accordingly (McKerrow et al., 2023; Linker et al., 2020). Otherwise, MORE-RNAseq will be useful for the majority of currently deposited RNA-seq data, as they do not necessarily contain the specific procedures for making libraries, such as the enrichment of 5′UTR of L1s or the poly-A depending method. For the other detection tools of L1 expression, as-is RepeatMasker entries are employed without curating the divided entries of full-length L1s and/or the monomer regions, causing unsuitable calculations and multimapping. For example, mouse fliL1-ID2022 is the same site as ID2022 on L1Base2, which shows no complete location information because of missed and divided entries by RepeatMasker (http://l1base.charite.de/details.php?DBN=mmflil1_8438&UID=2022). In MORE reference, these have been curated and available for use. Therefore, the MORE reference has advantages, including curated L1 information for analysis.

For our pipeline, we used STAR and RSEM for mapping and read count and R for statistical analyses and visualization, which are independently changeable based on the purpose of the user. It should be noted that some aligners limited the maximum number of mappable reads at one site, making it impossible to estimate expression from multicopy genes or elements. We used STAR as an aligner and set the values of several options as large ones that do not practically limit the computation (--outFilterMultimapNmax 100,000, --outSAMprimaryFlag AllBestScore, and--outSAMmultNmax −1 is our typical settings of STAR for MORE-RNAseq, for example). There are limitations regarding actively expressed non-rc-L1s and population-specific rc-L1s, which merit further attention and analysis. Development of curated reference set and customizable pipeline will present a valuable resource for the scientific community.

## Data Availability

The datasets presented in this study can be found in online repositories. The names of the repository/repositories and accession number(s) can be found in the article/Supplementary Material.

## References

[B1] Adey N. B. Schichman S. A. Hutchison C. A. Edgell M. H. (1991). Composite of A and F-type 5’ terminal sequences defines a subfamily of mouse LINE-1 elements. J. Mol. Biol. 221, 367–373. 10.1016/0022-2836(91)80057-2 1920423

[B2] Ansaloni F. Gualandi N. Esposito M. Gustincich S. Sanges R. (2022). TEspeX: consensus-specific quantification of transposable element expression preventing biases from exonized fragments. Bioinformatics 38, 4430–4433. 10.1093/bioinformatics/btac526 35876845 PMC9477521

[B3] Beck C. R. Garcia-Perez J. L. Badge R. M. Moran J. V. (2011). LINE-1 elements in structural variation and disease. Annu. Rev. Genomics Hum. Genet. 12, 187–215. 10.1146/annurev-genom-082509-141802 21801021 PMC4124830

[B4] Bundo M. Toyoshima M. Okada Y. Akamatsu W. Ueda J. Nemoto-Miyauchi T. (2014). Increased l1 retrotransposition in the neuronal genome in schizophrenia. Neuron 81, 306–313. 10.1016/j.neuron.2013.10.053 24389010

[B5] Criscione S. W. Zhang Y. Thompson W. Sedivy J. M. Neretti N. (2014). Transcriptional landscape of repetitive elements in normal and cancer human cells. BMC Genomics 15, 583. 10.1186/1471-2164-15-583 25012247 PMC4122776

[B6] De C. M. Ito T. Petrashen A. P. Elias A. E. Skvir N. J. Criscione S. W. (2019). L1 drives IFN in senescent cells and promotes age-associated inflammation. Nature 566, 73–78. 10.1038/s41586-018-0784-9 30728521 PMC6519963

[B7] Deininger P. Morales M. E. White T. B. Baddoo M. Hedges D. J. Servant G. (2017). A comprehensive approach to expression of L1 loci. Nucleic Acids Res. 45, e31. 10.1093/nar/gkw1067 27899577 PMC5389711

[B8] Guler G. D. Tindell C. A. Pitti R. Wilson C. Nichols K. KaiWai Cheung T. (2017). Repression of stress-induced LINE-1 expression protects cancer cell subpopulations from lethal drug exposure. Cancer Cell 32, 221–237.e13. 10.1016/j.ccell.2017.07.002 28781121

[B9] Jin Y. Tam O. H. Paniagua E. Hammell M. (2015). TEtranscripts: a package for including transposable elements in differential expression analysis of RNA-seq datasets. Bioinformatics 31, 3593–3599. 10.1093/bioinformatics/btv422 26206304 PMC4757950

[B10] Kazazian H. H. (2000). L1 retrotransposons shape the mammalian genome. Science 289, 1152–1153. 10.1126/science.289.5482.1152 10970230

[B11] Kumari R. Jat P. (2021). Mechanisms of cellular senescence: cell cycle arrest and senescence associated secretory phenotype. Front. Cell Dev. Biol. 9, 645593. 10.3389/fcell.2021.645593 33855023 PMC8039141

[B12] Lagerwaard B. Nieuwenhuizen A. G. Bunschoten A. de Boer V. C. Keijer J. (2021). Matrisome, innervation and oxidative metabolism affected in older compared with younger males with similar physical activity. J. Cachexia Sarcopenia Muscle 12, 1214–1231. 10.1002/jcsm.12753 34219410 PMC8517362

[B13] Lander E. S. Linton L. M. Birren B. Nusbaum C. Zody M. C. Baldwin J. (2001). Initial sequencing and analysis of the human genome. Nature 409, 860–921. 10.1038/35057062 11237011

[B14] Lerat E. Fablet M. Modolo L. Lopez-Maestre H. Vieira C. (2017). TEtools facilitates big data expression analysis of transposable elements and reveals an antagonism between their activity and that of piRNA genes. Nucleic Acids Res. 45, e17. 10.1093/nar/gkw953 28204592 PMC5389681

[B15] Linker S. B. Randolph-Moore L. Kottilil K. Qiu F. Jaeger B. N. Barron J. (2020). Identification of *bona fide* B2 SINE retrotransposon transcription through single-nucleus RNA-seq of the mouse hippocampus. Genome Res. 30, 1643–1654. 10.1101/gr.262196.120 33122305 PMC7605253

[B16] McKerrow W. Kagermazova L. Doudican N. Frazzette N. Kaparos E. Evans S. A. (2023). LINE-1 retrotransposon expression in cancerous, epithelial and neuronal cells revealed by 5’ single-cell RNA-Seq. Nucleic Acids Res. 51, 2033–2045. 10.1093/nar/gkad049 36744437 PMC10018344

[B17] Waterston R. H. Lindblad-Toh K. (2002). Initial sequencing and comparative analysis of the mouse genome. Nature 420, 520–562. 10.1038/nature01262 12466850

[B18] Novototskaya-Vlasova K. A. Neznanov N. S. Molodtsov I. Hall B. M. Commane M. Gleiberman A. S. (2022). Inflammatory response to retrotransposons drives tumor drug resistance that can be prevented by reverse transcriptase inhibitors. Proc. Natl. Acad. Sci. U. S. A. 119, e2213146119. 10.1073/pnas.2213146119 36449545 PMC9894111

[B19] Ostertag E. M. Kazazian H. H. (2001). Biology of mammalian L1 retrotransposons. Annu. Rev. Genet. 35, 501–538. 10.1146/annurev.genet.35.102401.091032 11700292

[B20] Patro R. Duggal G. Love M. I. Irizarry R. A. Kingsford C. (2017). Salmon provides fast and bias-aware quantification of transcript expression. Nat. Methods 14, 417–419. 10.1038/nmeth.4197 28263959 PMC5600148

[B21] Penzkofer T. Jäger M. Figlerowicz M. Badge R. Mundlos S. Robinson P. N. (2017). L1Base 2: more retrotransposition-active LINE-1s, more mammalian genomes. Nucleic Acids Res. 45, D68–D73. 10.1093/nar/gkw925 27924012 PMC5210629

[B22] Pickeral O. K. Makałowski W. Boguski M. S. Boeke J. D. (2000). Frequent human genomic DNA transduction driven by LINE-1 retrotransposition. Genome Res. 10, 411–415. 10.1101/gr.10.4.411 10779482 PMC310862

[B23] Simon M. Van Meter M. Ablaeva J. Ke Z. Gonzalez R. S. Taguchi T. (2019). LINE1 derepression in aged wild-type and SIRT6-deficient mice drives inflammation. Cell Metab. 29, 871–885.e5. 10.1016/j.cmet.2019.02.014 30853213 PMC6449196

[B24] Sookdeo A. Hepp C. M. McClure M. A. Boissinot S. (2013). Revisiting the evolution of mouse LINE-1 in the genomic era. Mob. DNA 4, 3. 10.1186/1759-8753-4-3 23286374 PMC3600994

[B25] St Laurent G. Hammell N. McCaffrey T. A. (2010). A LINE-1 component to human aging: do LINE elements exact a longevity cost for evolutionary advantage? Mech. Ageing Dev. 131, 299–305. 10.1016/j.mad.2010.03.008 20346965 PMC2875337

[B26] Streva V. A. Jordan V. E. Linker S. Hedges D. J. Batzer M. A. Deininger P. L. (2015). Sequencing, identification and mapping of primed L1 elements (SIMPLE) reveals significant variation in full length L1 elements between individuals. BMC Genomics 16, 220. 10.1186/s12864-015-1374-y 25887476 PMC4381410

[B27] Watanabe R. Nakachi Y. Matsubara H. Ueda J. Ishii T. Ukai W. (2023). Identification of epigenetically active L1 promoters in the human brain and their relationship with psychiatric disorders. Neurosci. Res. 195, 37–51. 10.1016/j.neures.2023.05.001 37141946

[B28] Yang W. R. Ardeljan D. Pacyna C. N. Payer L. M. Burns K. H. (2019). SQuIRE reveals locus-specific regulation of interspersed repeat expression. Nucleic Acids Res. 47, e27. 10.1093/nar/gky1301 30624635 PMC6411935

