# Supplementary material for: MORE-RNAseq: a pipeline for quantifying retrotransposition-capable LINE1 expression based on RNA-seq data
Source: Front Bioinform. 2025 May 22;5:1575346. doi: 10.3389/fbinf.2025.1575346 (PMC12138260; doi:10.3389/fbinf.2025.1575346)

Legends to Supplementary Figures

Supplementary Figure 1. All L1 sequences were manually curated, and L1-specific regions were chosen as the reference.

(**A**) The regions chosen as the human and mouse MORE reference are shown as boxes. Because some L1s are divided into multiple entries as different L1 annotations or no entries partially in RepeatMasker and L1Base 2 (fli-L1s: full-length intact L1s), and almost all rc-L1s are located in surrounding repetitive sequences, the reference regions of rc-L1s were curated and selected carefully. For human rc-L1s, because of their well-conserved structures, we adopted from 5' termini of L1 entries itself to the nearest polyA signals downstream of each ORF2 as the L1 reference region. In the case of the mouse MORE reference, the criteria of reference choice were generally the same as for humans. However, many subtypes of L1 are involved, so those structures vary. The 3'-termini of mouse L1s were confirmed manually, and almost all mouse L1s have similar structures from the ORF2 end to the nearest polyA signals, except for pseudopolyA signals and/or longer additional 3' tails. Therefore, a 3' cutoff at the 665 bp position from the ORF2 end was adopted for all mouse reference L1 sequences. Additionally, we adopted the 5' cutoff at -195 bp from the ORF1 start site to exclude the monomer repeat region from the mouse reference to avoid the massive amount of incorrect mapping of reads derived from repetitive sequences other than L1s. (**B**) Alignment of all 146 rc-L1s used as MORE references for humans. In the left panel, the plot shows that all full-length intact L1s align with the ORF1 start position as zero, which is shown as dashed lines. In the right panel, the plot shows the alignment with the ORF2 end position as zero. L1 entries covering both ORF1 and ORF2 are shown as 'covering ORFs'; for the divided L1 entries, the 5' side, the 3' side and the inside of ORF1/ORF2 regions are shown as '5'+ORFs', '3'+ORFs', and

'inside ORFs', respectively. (**C**) Alignment of all 2811 rc-L1s used as MORE references for mice. In the mouse alignment, the 5' cutoff position in the left panel is shown as a line at the -195 bp position from the ORF1 start position, and the 3' cutoff position in the right panel is shown as a line at the 665 bp position from the ORF2 end position. L1 entries covering both ORF1 and ORF2 are shown as 'covering ORFs'; for the divided L1 entries, the 5' side, 3' side and inside of ORF1ORF2 regions are shown as '5'+ORFs', '3'+ORFs', and 'inside ORFs', respectively. All ID numbers of L1 in **B** and **C** are consistent with the IDs of L1Base2 entries.

Supplementary Figure 2. Results comparison between MORE-RNAseq and TEtranscripts (GSE100751).

Comparison of results derived from MORE-RNAseq and TEtranscripts using either the same or different reference files. (A, D) With MORE-RNAseq, an increase in overall rc-L1 expression was observed in the carboplatin ($P = 0.026$, Welch's t-test) and erlotinib ($P = 0.022$) groups compared to the DMSO group. (B, E) Using TEtranscripts with its default GTF reference, a significant increase in overall L1 expression was found in the carboplatin group ($P = 0.019$) and a trend towards increase in the erlotinib group ($P = 0.077$). (C, F) With TEtranscripts and the GTF reference from MORE-RNAseq, an increase in overall rc-L1 expression was observed in the carboplatin ($P = 0.035$) and erlotinib ($P = 0.001$) groups.

Supplementary Figure 1

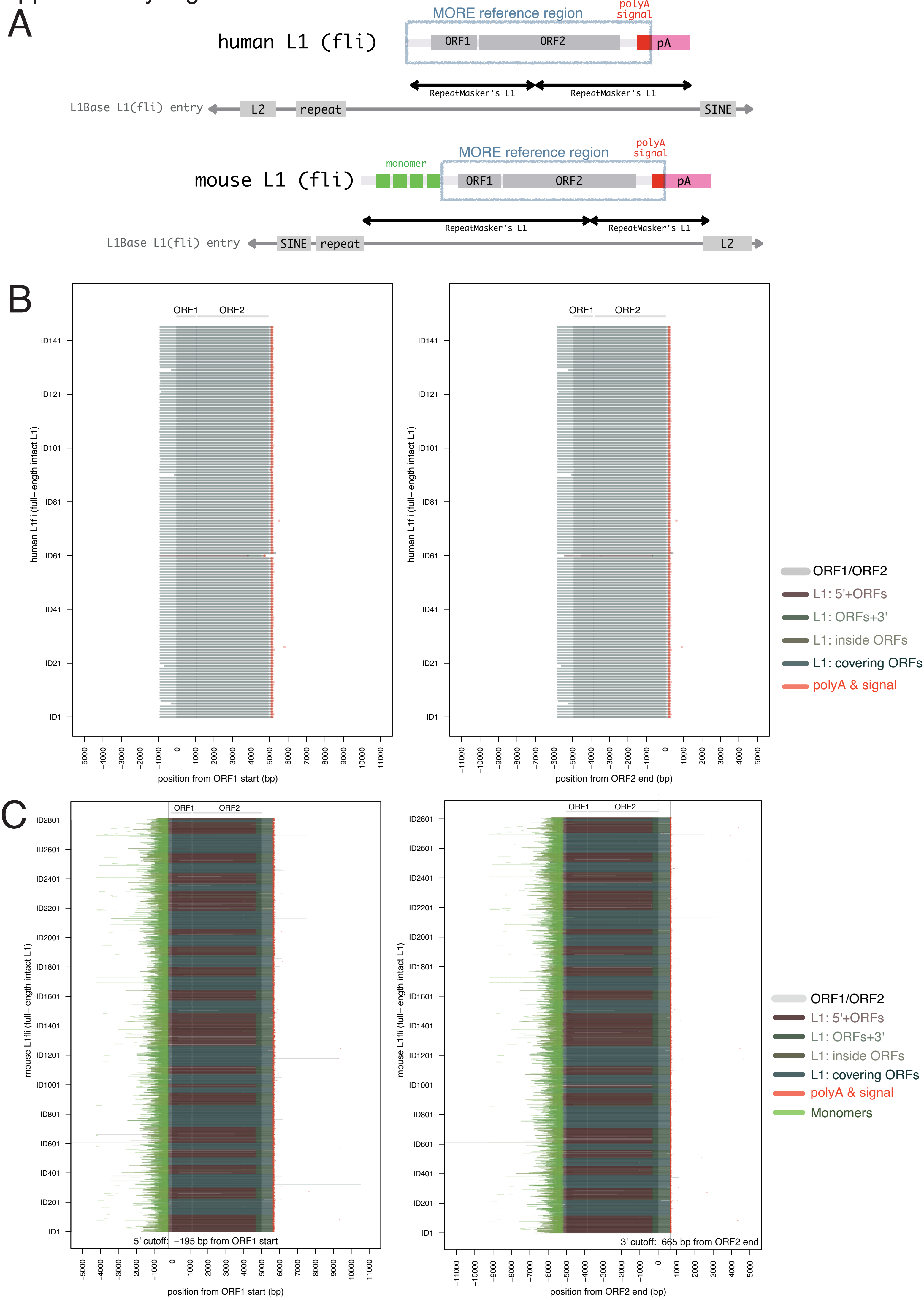

Supplementary Figure 2

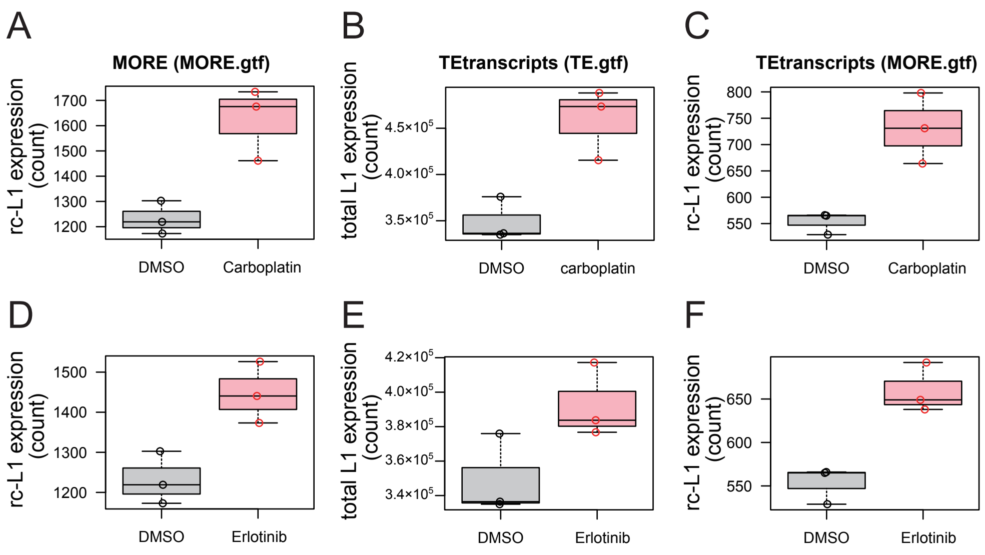

Supplement: Supplementary file 1 [file DataSheet1.pdf]
